# Endocrine Disruption in Freshwater Cladocerans: Transcriptomic Network Perspectives on TBOEP and PFECHS Impacts in *Daphnia magna*

**DOI:** 10.3390/ijms262412146

**Published:** 2025-12-17

**Authors:** Hyun Woo Kim, Seok-Gyu Yun, Ju Yeon Park, Jun Lee, Jun Pyo Han, Dong Yeop Shin, Jong Hun Lee, Eun-Min Cho, Young Rok Seo

**Affiliations:** 1 Department of Life Science, Biomedi Campus, Institute of Environmental Medicine for Green Chemistry, Dongguk University, 32 Dongguk-ro, Ilsandong-gu, Goyang-si 10326, Republic of Korea; 2 Department of Nano, Chemical & Biological Engineering, College of Natural Science and Engineering, Seokyeong University, Seoul 02173, Republic of Korea; 3 Department of Food Science and Biotechnology, Gachon University, Seongnam 13120, Republic of Korea

**Keywords:** *Daphnia magna*, endocrine disruption, TBOEP, PFECHS, transcriptomic analysis, adverse outcome pathway (AOP)

## Abstract

Freshwater cladocerans such as *Daphnia magna* (*D. magna*) are keystone grazers whose hormone-regulated life history traits make them sensitive sentinels of endocrine-disrupting chemicals (EDCs). The organophosphate flame-retardant tris(2-butoxyethyl) phosphate (TBOEP) and perfluoroethylcyclohexane sulfonate (PFECHS) now co-occur at ng L^−1^–µg L^−1^ in surface waters, yet their chronic sub-lethal impacts on invertebrate endocrine networks remain unclear. We analysed two publicly available 21-day microarray datasets (TBOEP: GSE55132; PFECHS: GSE75607) using gene ontology enrichment, STRING protein interaction networks, Drosophila phenotype mapping, and KEGG (Kyoto Encyclopaedia of Genes and Genomes)-anchored frameworks to build putative adverse outcome pathways (AOPs) for *D. magna*. Differentially expressed genes were clustered into functional modules and hub nodes were ranked by degree and betweenness. TBOEP suppressed moulting and growth, altering 1157 genes enriched for metabolism and membrane processes; hubs VRK1, MIB2, and adenylosuccinate synthetase formed a muscle anatomical development sub-network. PFECHS down-regulated vitellogenin and shifted 879 genes dominated by oxidative-stress and glutathione-metabolism signatures; central nodes UBC9, eIF4A-III, Tra-2α, and HDAC1 linked meiotic-cycle, oogenesis, and cyclic-compound binding. Despite chemical dissimilarity, both compounds converged on Wnt-signalling nodes—TBOEP via presenilin-1, and PFECHS via CK1ε/CK2—thereby reducing TCF/LEF-dependent transcription. Predicted outcomes include impaired oocyte maturation, reduced fecundity, and stunted body size, consistent with observed decreases in length and vitellogenin protein. Our network analysis, based on high-dose, sub-lethal exposures used in the underlying microarray studies, indicates that TBOEP- and PFECHS-induced perturbations can destabilise endocrine, developmental, and metabolic pathways in *D. magna* without overt lethality, and highlights Wnt-centred key events and hub genes as candidate biomarkers to be evaluated in future low-dose studies that use environmentally realistic exposure scenarios. Hub genes and Wnt-mediated key events emerge as sensitive biomarkers for monitoring mixed EDC exposure.

## 1. Introduction

Endocrine-disrupting chemicals (EDCs) are exogenous substances that alter hormone synthesis, metabolism, transport, or receptor signalling, thereby impairing growth, development, and reproduction in exposed organisms [1]. Notably, freshwater ecosystems receive a continuous influx of industrial effluent, agricultural runoff, and municipal wastewater, exposing aquatic species to complex EDC mixtures [2,3]. Although early research focused on vertebrates, classic field observations, such as tributyltin-induced imposex in marine gastropods, have demonstrated that invertebrate taxa are equally vulnerable and that endocrine disruption can drive population-level changes [4].

*Daphnia magna* (*D. magna*) is a widely used sentinel for detecting endocrine disruption. The lifecycle of *D. magna* is exquisitely hormone-dependent, with juvenile hormones regulating the transition from clonal to sexual reproduction, while ecdysteroids control moulting and somatic growth. Consequently, shifts in sex ratio, fecundity, or moulting frequency provide sensitive read-outs of endocrine perturbation [5,6]. Owing to its well-characterised life history, short generation time, and extensive genomic resources, *D. magna* is routinely used to screen chemicals that target invertebrate hormonal pathways. In addition to classic flame retardants and PFAS, recent reviews highlight that emerging contaminants such as micro- and nanoplastics can also trigger endocrine-active responses in aquatic organisms, including altered steroidogenesis, vitellogenin expression, and gonadal development [7,8].

**Figure 1:**
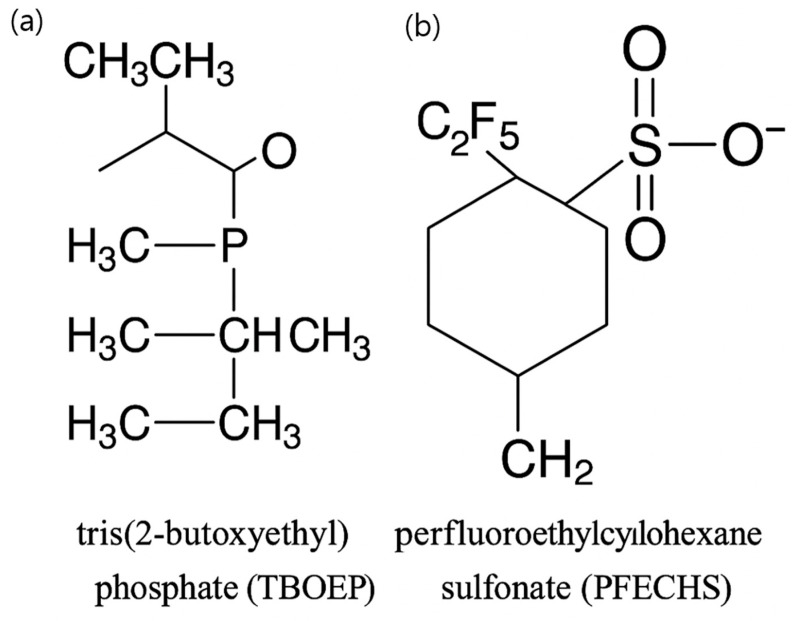
Chemical structures of tris(2-butoxyethyl) phosphate (TBOEP) and perfluoro ethylcyclohexane sulfonate (PFECHS). (**a**) TBOEP is a neutral organophosphate triester bearing three 2-butoxyethyl chains attached to a central phosphate group, which is a motif typical of high-production-volume flame retardants and plasticizers. (**b**) PFECHS is an anionic perfluoroalkyl sulfonate comprising a perfluoroethyl group and a sulfonate moiety linked to a cyclohexane ring, characteristic of highly persistent PFAS. These distinct functional groups and polarities underlie differences in hydrophobicity, bioaccumulation potential, and protein binding, yet both structures are compatible with interactions at endocrine-related molecular targets.

Among the many contaminants detected in surface waters, two compounds warrant scrutiny: tris(2-butoxyethyl) phosphate (TBOEP), a high-production-volume organophosphate flame retardant that is now ubiquitously identified at microgram-per-litre levels [9]. Chronic exposure of *D. magna* to TBOEP concentrations (≈10 µg L^−1^) dysregulates transcripts in juvenile-hormone and ecdysteroid pathways, lowers moulting frequency, and retards growth without causing acute lethality [10]. Studies in zebrafish have revealed altered hypothalamic–pituitary–gonadal gene expression, elevated male estradiol levels, and reduced fecundity, underscoring the cross-phylum endocrine activity of TBOEP [11]. Perfluoroethylcyclohexane sulfonate (PFECHS), used in aviation hydraulic fluids, also persists in remote waters at nanogram-per-litre concentrations [12]. The contrasting chemistries of these two compounds are illustrated in Figure 1. TBOEP is a neutral organophosphate triester with three 2-butoxyethyl chains, whereas PFECHS is an anionic perfluoroalkyl sulfonate bearing a perfluoroethyl group and a sulfonate moiety on a cyclohexane ring. These distinct functional groups and polarities imply different environmental fates and bioaccumulation patterns, yet both structures are compatible with interactions at endocrine-related molecular targets (Figure 1).

Adverse outcome pathway (AOP) frameworks provide a structured means to link a molecular-initiating event through successive key events to apical outcomes that are ecologically relevant and regulatory ready [13,14]. Integrating high content transcriptomics with AOP thinking allows early mechanistic signals—such as Wnt/β catenin perturbation [15]—to be traced to reproductive endpoints in cladocerans.

The pervasive occurrence of TBOEP and the extreme persistence of PFECHS suggest that *D. magna* populations experience chronic, low-level exposure capable of disrupting endocrine homeostasis and, by extension, freshwater ecosystem function. A 12-day exposure of *D. magna* to sub-milligram levels markedly suppressed vitellogenin mRNA and protein while up-regulating cuticle-synthesis genes, indicating sub-lethal endocrine interference without immediate effects on survival or reproduction [16]. Even at low ambient concentrations, evidence of endocrine-mediated effects at higher doses exists, raising concern about cumulative or mixture-driven impacts. Since *D. magna* influences water-column clarity and serves as prey for higher trophic levels, any impairment of its reproduction or development could cascade through planktonic communities. Therefore, clarifying the molecular mechanisms, effects, and consequences of TBOEP and PFECHS exposure is critical for robust ecological risk assessment and informed chemical management. The present study integrates public transcriptomic datasets, gene ontology enrichment, interaction network analysis, and adverse-outcome-pathway construction to elucidate how chronic exposure to these two EDCs reshapes endocrine-regulated processes in *D. magna*. We integrate public 21-day transcriptomic datasets generated at high nominal concentrations of TBOEP (1470 µg L^−1^) and PFECHS (6 mg L^−1^) as mechanistic “stress tests” to probe susceptible endocrine and developmental pathways in *D. magna*, rather than as environmentally realistic exposure scenarios.

## 2. Results

### 2.1. TBOEP

#### 2.1.1. TBOEP Toxicity and Transcriptomic Insights

**Figure 2:**
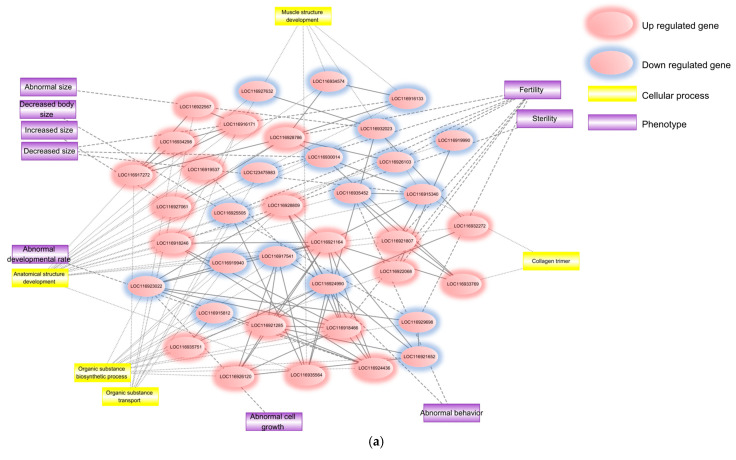

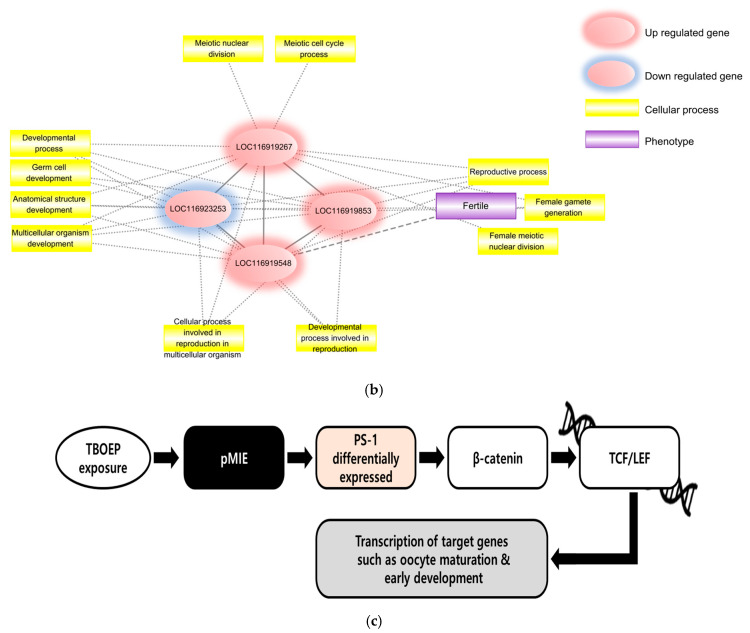
Biological network and AOP overview of chronic TBOEP toxicity in *D. magna*. (**a**) Integrated biological network derived from DEGs after long-term TBOEP exposure, highlighting the tight coupling between developmental and reproductive adverse effects. (**b**) Endocrine-centred sub-network extracted from the same DEG set, underscoring strong links to female reproductive processes and ontogeny. Red nodes denote up-regulated genes, and blue nodes denote down-regulated genes. Yellow rectangles mark associated cellular-process terms; purple rectangles indicate phenotype annotations. Solid blue edges represent gene–gene associations; solid grey edges connect genes to phenotypes, and dashed grey edges connect genes to cellular processes. (**c**) Proposed adverse-outcome pathway (AOP) describing the sequence of key events triggered by chronic TBOEP exposure.

Additional mechanistic insight into TBOEP toxicity was obtained by analysis of the public microarray dataset GSE55132, which profiles whole-body RNA from *D. magna* exposed to 1470 µg L^−1^ TBOEP for 21 days; this nominal concentration is several orders of magnitude higher than typical ng L^−1^–low µg L^−1^ detections in surface waters and should therefore be interpreted as a high-dose mechanistic exposure rather than an environmentally realistic scenario. Differentially expressed genes (DEGs) were extracted, followed by gene ontology (GO) enrichment, interaction network synthesis, and formulation of a putative adverse outcome pathway (AOP). The resulting GO terms, DEG-centred protein network, and AOP framework together outline how chronic TBOEP exposure perturbs metabolic processes, membrane-associated cellular components, and hormone-regulated developmental pathways (Figure 2). 

#### 2.1.2. GO Enrichment in TBOEP Exposure

The top 10 activated and suppressed biological processes (BPs), cellular components (CCs), and molecular functions (MFs) were identified at a false discovery rate (FDR) < 0.05. For BPs, ten activated and six suppressed terms were observed, with the ontology “cellular process” present in both lists. Activated BPs were predominantly associated with metabolic-process-related GO terms, whereas among the suppressed BPs’ “positive regulation of transport” was identified (Table 1). Among the ten activated and five suppressed statistically significant CCs, ontologies including “cellular anatomical entity”, “cytoplasm”, “intercellular anatomical structure”, and “intracellular organelle” were concurrently identified. In the activated CCs, GO terms related to anatomical entities and membranes were observed, and similar ontologies related to anatomical entities and membranes were also present in the suppressed CCs (Table 1). Statistically significant MFs comprised five activated and one suppressed term. The activated MFs primarily included catalytic-activity- and binding-related ontologies, whereas the suppressed MF was associated with binding (Table 1). In summary, GO analysis indicated that chronic TBOEP exposure primarily affected metabolic biological processes, transport regulation, cellular anatomical entities and membranes, and catalytic and binding molecular functions. A subset of endocrine-related GO categories derived from the main network included organic substance biosynthetic process, organic substance transport, anatomical structure development, muscle structure development, and collagen trimer (Table 2).

#### 2.1.3. TBOEP Functional Network Evidence

To gain insight into key DEGs in TBOEP-exposed *D. magna*, protein–protein interactions (PPIs) were retrieved from STRING-DB and used to construct a PPI network (Figure 2a). These interactions clarify how numerous DEGs inter-connect at the molecular level. The proteins encoded by DEGs, regardless of expression direction, were jointly subjected to GO analysis, which highlighted terms linked to the synthesis and transport of organic substances, developmental processes, and collagen trimers (Table 1).

For a more comprehensive view of TBOEP toxicity, a biological network integrating protein–protein, protein–cell process, and protein–orthologous phenotype relationships were built (Figure 2a). Five statistically significant (FDR < 0.05) GO terms associated with endocrine disruption were recovered among biological processes, whereas no significant terms were found for cellular components or molecular functions in this endocrine-focused subset. To predict phenotypes, DEGs were first matched to *Drosophila melanogaster* orthologues using BLASTp v2.17.0 because phenotype–protein links are sparse for *D. magna*. The well-annotated fruit-fly dataset enabled inference of size- and reproduction-related phenotypes via FlyBase.

Central genes were mined from the STRING network (Figure 2a) using edge degree and betweenness centrality scores. Six hubs emerged: LOC116918246 (serine/threonine-protein kinase VRK1), LOC116935564 (E3 ubiquitin–protein ligase MIB2), LOC116924990 (adenylosuccinate synthetase), LOC116918466 (28S ribosomal protein S2, mitochondrial), LOC116921285 (tyrosine-tRNA ligase, cytoplasmic), and LOC116917541 (glutamine synthetase). A condensed network (Figure 2b) linking these hubs to cell-process and orthologous phenotype nodes highlighted associations with anatomical and muscle structure development, reproduction, and infertility.

#### 2.1.4. TBOEP Putative AOP Analysis

A putative adverse outcome pathway (AOP) for TBOEP exposure in *D. magna* was derived from the KEGG Wnt-signalling pathway (Figure 2c). In this scheme, expression changes in PS-1 following chronic TBOEP exposure were treated as proximal molecular perturbation. The altered expression of PS-1 was associated with changes in β-catenin levels, thereby impacting members of the TCF/LEF transcription-factor family. These three biological events were regarded as key events (KEs), and the downstream adverse outcome (AO) was described as altered transcription of target genes involved in oocyte maturation and early development.

Overall, we identified DEGs from whole-body transcriptomes of *D. magna* exposed to TBOEP for 21 days and characterised their functional attributes using GO enrichment, protein–protein interaction networks, orthologous phenotypes, and a Wnt-based putative AOP (Figure 2).

### 2.2. PFECHS

#### 2.2.1. Transcriptomic Dataset and Overall Response

The GSE75607 dataset, deposited in a public database, consists of gene expression data evaluating the reproductive performance in *D. magna* following chronic exposure to PFECHS. The gene expression experiment conducted by Houde M. et al., following OECD guidelines [16,17], encompasses the adverse effects of chronic exposure to 6 mg L^−1^ PFECHS for 21 days; as with the TBOEP dataset, this nominal concentration is several orders of magnitude higher than environmental detections and should therefore be considered a high-dose mechanistic exposure rather than an environmentally realistic scenario. From this dataset, DEGs were extracted and used to interpret the chronic toxicity effects of PFECHS through GO enrichment, network construction, and the development of a putative AOP.

#### 2.2.2. GO Enrichment in PFECHS Exposure

To elucidate the cellular significance of DEGs elicited by chronic PFECHS exposure, GO profiles of activated and suppressed transcripts were examined. Activated or suppressed DEGs were assigned to biological processes (BPs), cellular components (CCs), and molecular functions (MFs); ontologies with an FDR < 0.05 were retained, and the ten most significant terms per category were collated (Table 3).

Among the ten activated and ten suppressed BPs, metabolic-process-related terms were dominant in both lists. “Cellular process”, “metabolic process”, “organic substance metabolic process”, “nitrogen compound metabolic process”, and “primary metabolic process” appeared in common. Excluding these metabolic terms, activated BPs included “response to stimulus”, “regulation of biological quality”, “glutathione metabolic process”, and “response to ethanol”, whereas suppressed BPs uniquely contained “biological regulation”, “cellular component organisation or biogenesis”, and “regulation of biological process” (Table 3).

Statistically significant activated or suppressed CCs were identified based on the top 10 ontologies with the lowest FDR (Table 3). Among these, “cellular anatomical entity”, “cytoplasm”, “intracellular anatomical structure”, “organelle”, and “intracellular organelle” were common to both activated and suppressed sets. Activated CCs were enriched for fibre-, organelle-, and extracellular-structure-related terms, including “sarcomere”, “supramolecular fibre”, and “Z disc”, whereas suppressed CCs highlighted “protein-containing complex”, “ribonucleoprotein complex”, “membrane-bounded organelle”, and related categories (Table 3).

In both activated and suppressed MFs, “catalytic activity”, “ion binding”, “binding”, and “protein binding” were common. Unique activated MFs were detoxification-related, such as “oxidoreductase activity”, “glutathione transferase activity”, and additional transferase activities, while unique suppressed MFs were largely binding-related (e.g., “organic cyclic compound binding” and “nucleic acid binding”) (Table 3). Overall, GO analysis indicated that PFECHS exposure extensively affected metabolic processes, cell-structure-related components and catalytic and binding molecular functions. Endocrine-related GO categories in the main network included organic substance biosynthetic process, developmental and reproductive processes, female gamete generation, organic substance transport, meiotic cell cycle processes, and organic cyclic compound binding (Table 4).

#### 2.2.3. PFECHS Functional Network Evidence

For a comprehensive understanding of PFECHS toxic mechanisms in *D. magna*, a biological network was constructed using PPIs, cell process relationships, and orthologous phenotypes. PPIs were collected from STRING-DB for *D. magna* and used to build a network capturing the reproductive toxicity of PFECHS.

**Figure 3:**
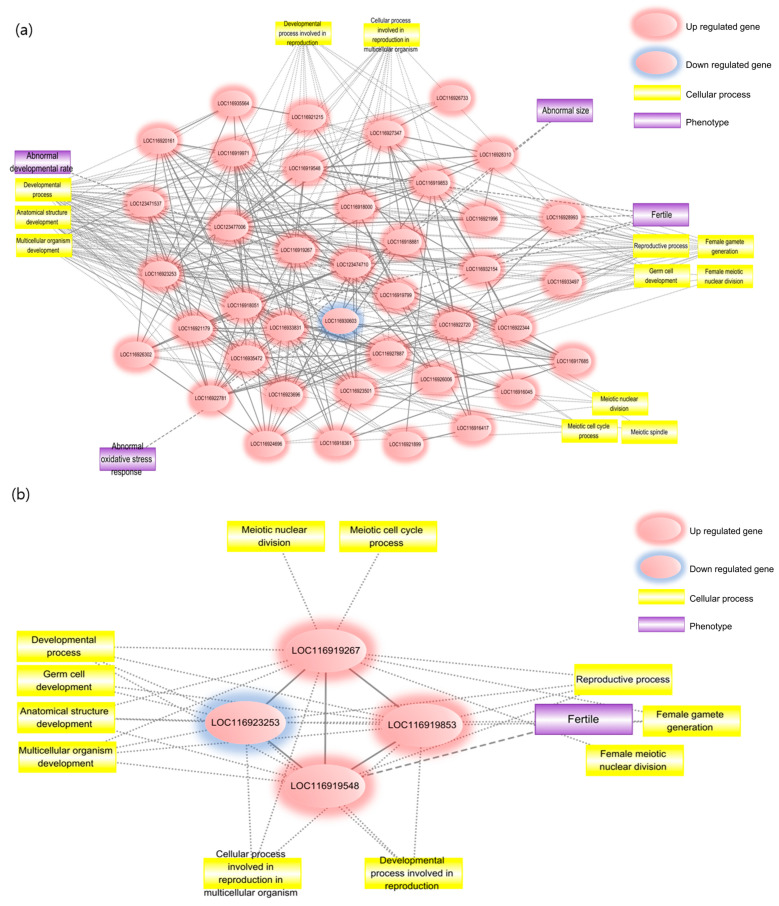

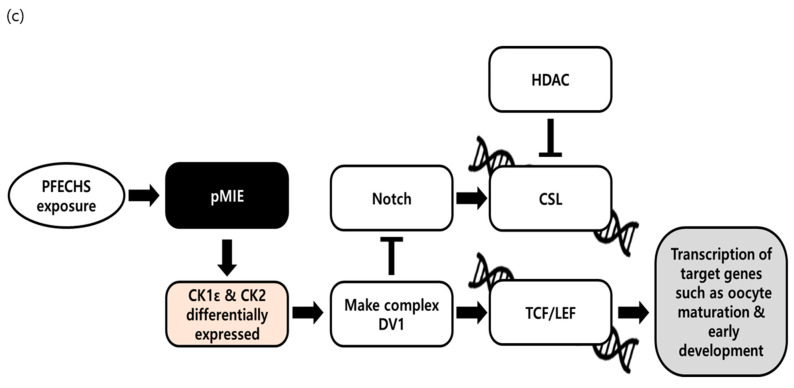
Biological network and AOP overview of chronic PFECHS toxicity in *D. magna*. (**a**) Integrated biological network derived from DEGs after long-term PFECHS exposure, showing the close inter-relationship between developmental and reproductive adverse effects. (**b**) Endocrine-focused sub-network extracted from the same DEG set, emphasising strong links to female reproductive processes and ontogeny. Red nodes indicate up-regulated genes; blue nodes indicate down-regulated genes. Yellow rectangles denote associated cellular-process terms, and purple rectangles denote phenotype annotations. Solid blue lines represent gene–gene associations; solid grey lines link genes to phenotypes, while dashed grey lines link genes to cellular processes. (**c**) Proposed adverse-outcome pathway (AOP) illustrating the sequence of key events triggered by chronic PFECHS exposure.

The biological network consists of PPIs, protein–cell process relationships, and protein–orthologous phenotype relationships (Figure 3a). The proteins constituting the network are encoded by DEGs, with their expression changes mirroring those of the DEGs. To elucidate network functions at the cellular level, GO ontologies were identified among DEG-encoding proteins, regardless of expression pattern. After confirming statistically significant (FDR < 0.05) gene ontologies associated with reproduction, a total of 14 ontologies were identified in BPs, while one each was found in CC and MF. Among the BPs, development-, reproduction-, and organic-substance-metabolism-related ontologies were prominent, whereas ontologies associated with cell division were identified in CC and organic compound binding in MF.

To infer phenotypes based on DEGs in *D. magna*, translated proteins of DEGs were converted to *D. melanogaster* proteins using NCBI BLASTp. For *D. magna*, information on phenotype-related proteins is limited, whereas for an evolutionary proximate species such as *D. melanogaster*, extensive phenotypic research has generated abundant data [18,19]. We identified phenotypes associated with reproduction and development in *D. melanogaster* from FlyBase for the proteins encoded by DEGs, obtaining orthologous phenotypes including abnormal developmental rate, abnormal size, altered fertility, and abnormal oxidative stress response. To identify central genes in the reproductive and developmental toxicity network of PFECHS, each gene in the PPI network (Figure 3b) was assessed based on edge degree and betweenness centrality. LOC116919267 (SUMO-conjugating enzyme UBC9), LOC116919853 (eukaryotic initiation factor 4A-III), LOC116919548 (transformer-2 protein homologue α), and LOC116923253 (histone deacetylase 1) displayed high centrality. Based on these central proteins, a condensed network (Figure 3c) was constructed using interactions among central proteins and between central proteins and cellular processes and orthologous phenotypes. In this condensed network, reproductive-related phenotypes such as fertility- and reproductive-related cellular processes, particularly “female gamete generation” and “female meiotic nuclear division”, were prominent.

#### 2.2.4. PFECHS Putative AOP Analysis

The putative AOP for PFECHS exposure in *D. magna* (Figure 3c) was outlined from the KEGG Wnt-signalling pathway. In the absence of a definitive molecular-initiating event (MIE) resolved directly from the transcriptomic data, the model postulates an initial perturbation of CK1ε and CK2 expression as a putative MIE. Altered CK1ε/CK2 levels are predicted to remodel the dishevelled (DVL) scaffold and, in turn, attenuate TCF/LEF transcriptional activity. These sequential changes constitute three key events (KEs), after which transcription of targets governing oocyte maturation and early development is altered. Downstream DEGs included vtg1, yolkless, and multiple cyclins, consistent with changes in yolk-protein uptake, nutrient provision, and cell cycle progression in developing oocytes. This AOP links CK1ε/CK2-mediated Wnt disruption to transcriptional changes in genes involved in female reproductive processes in *D. magna*.

## 3. Discussion

### 3.1. TBOEP—Integration with Toxicological Evidence

TBOEP is detected in surface waters worldwide, and its chronic presence is recognised as an ecological concern [9]. Multigenerational 21-day assays with *D. magna* report unchanged survival and brood production but significant reductions in body length and moulting frequency, accompanied by persistent transcriptional shifts in ecdysteroid and juvenile hormone pathways that control growth and reproduction [10]. Complementary vertebrate data strengthen the endocrine disruption signal: adult zebrafish maintained for 21 days at 5–500 µg L^−1^ produce fewer eggs, their progeny hatch and survive less often, and histology shows arrested oocyte maturation and delayed spermiation, coincident with elevated 17β-estradiol, altered testosterone, and broad disruption of hypothalamic–pituitary–gonadal transcripts [11]. Embryos exposed to 2–200 µg L^−1^ develop oedema and skeletal deformities, while hormone synthesis genes are suppressed and receptor genes are induced across thyroid and gonadal axes—confirming endocrine dysregulation during development [20]. Beyond endocrine effects, oxidative stress responses have also been recorded: in *D. magna*, catalase activity drops in F_1_ generation after 28 days at 20 µg L^−1^, glutathione-S-transferase and heat-shock-protein transcripts fall at low doses, and ABC-transporter genes rise only at milligram-per-litre levels [10]. These invertebrate and vertebrate studies collectively indicate that TBOEP can impair growth, reproduction, hormone signalling, and redox homeostasis at sub-lethal concentrations. It should be noted that the TBOEP and PFECHS concentrations used in the underlying microarray experiments (1470 µg L^−1^ and 6 mg L^−1^, respectively) are several orders of magnitude higher than typical ng L^−1^–low µg L^−1^ detections in surface waters; consequently, the AOPs developed here should be regarded as high-dose mechanistic hypotheses that require confirmation at lower, environmentally realistic concentrations.

Our transcriptomic analysis is consistent with this mode of action and refines it mechanistically. In crustaceans including Daphnia, reproductive output is tightly constrained by energy acquisition and allocation, and food- or metabolically stressed animals typically reduce clutch size, delay reproduction, or reallocate resources away from gamete production. In C. elegans, lipid metabolism is regulated by signals from the reproductive system [21], and in humans, successful reproduction is associated with tightly regulated energy metabolism [22]. Therefore, the predominance of metabolic process GO terms among activated BPs, together with suppression of transport-related processes, indirectly suggests that chronic TBOEP-induced metabolic abnormalities may affect the reproductive capacity of *D. magna*. At the cellular component level, both activated and suppressed CCs included anatomical entity and membrane-related ontologies, indicating abnormalities within cell components and cell membranes. Considering that damaged cells are replaced by new cells through apoptosis, persistent perturbation of these CCs by chronic TBOEP exposure could interfere with normal tissue renewal and, consequently, affect the developmental stages of *D. magna* offspring. From the perspective of TBOEP as an endocrine disruptor, binding-related ontologies can be interpreted as reflecting the binding of reproductive hormones to their receptors, whereas catalytic-activity-related ontologies may correspond to the enzymatic breakdown or synthesis of reproductive hormones. The combination of altered binding and catalytic functions therefore supports the view that TBOEP disrupts reproductive-hormone metabolism and signalling.

Within the PPI and integrated biological networks, endocrine-related GO categories such as organic substance biosynthetic process and organic substance transport are readily mapped to hormone synthesis and trafficking. Ontologies describing anatomical and muscle structure development imply that chronic TBOEP exposure during early life stages disrupts organogenesis, particularly of muscle. When *D. magna* muscles malfunction, animals experience restricted swimming, cardiac-rhythm abnormalities and impaired movement of the head, eyes, and other appendages [23,24,25]. These phenotypes are compatible with the reproduction- and size-related adverse effects identified via orthologous phenotypes in *D. melanogaster*. The hub genes highlighted in our TBOEP network further support a developmental and reproductive mode of action. VRK1 is broadly expressed across zebrafish tissues [26] and is essential for early embryogenesis in Drosophila [27]. MIB2 participates in post-translational regulation in all major eukaryotic lineages [28] and mediates ubiquitination to activate Notch signalling in zebrafish [29]. Adenylosuccinate synthetase fuels ATP synthesis under stress in *D. magna* [30] and influences vertebrate eye development. Together with glutamine synthetase and mitochondrial ribosomal proteins, these hubs point to the convergent disruption of energy metabolism, cell proliferation, and developmental signalling. The condensed network underscores this by linking central genes to anatomical and muscle structure development, reproduction, and infertility phenotypes. The Wnt-based putative AOP integrates these observations into a coherent sequence of key events. Because purely genetic analyses make it difficult to pinpoint a definitive molecular-initiating event (MIE) for TBOEP, we treated PS-1 expression changes as a proximal perturbation. Among the Wnt-pathway-related DEGs, PS-1 was selected as a candidate proximal perturbation because it showed consistent differential expression across contrasts, occupied a central position in the Wnt sub-network, and has been repeatedly implicated in β-catenin regulation and reproductive signalling in vertebrate models. Altered PS-1 can modify β-catenin stability and TCF/LEF activity, thereby reshaping transcriptional programmes that govern oocyte maturation and early development. Through this putative AOP, chronic TBOEP exposure is proposed to induce abnormal transcription of target genes involved in female reproduction and development in *D. magna*.

In summary, the combined GO, network, and AOP analyses suggest that chronic TBOEP exposure induces unusual metabolism, disrupts cell anatomical structures, decreases offspring developmental potential, and alters binding processes. These changes are consistent with adverse effects on reproduction, reproductive-hormone metabolism, and size-related phenotypes, and highlight central roles for genes such as LOC116918246, LOC116935564, and LOC116924990 in the chronic toxicity mechanism of TBOEP. We note that the canonical PS-1–β-catenin–TCF/LEF cascade has been most extensively characterised in vertebrates, and its application to *D. magna* at present represents a mechanistic extrapolation rather than a fully validated invertebrate pathway.

### 3.2. PFECHS—Integration with Toxicological Evidence

PFECHS is a cyclic PFAS used in aviation hydraulic fluids and has been measured in river water at low nanogram-per-litre concentrations [31], while its toxicology has only recently been described [12]. When *D. magna* were exposed for 12 days to 0.06–6 mg L^−1^, vitellogenin transcripts and protein content fell sharply while survival, moulting, and brood size remained unchanged, signalling an anti-estrogenic endocrine-disrupting action likely to impair oocyte provisioning if exposure persists [16]. Vertebrate evidence is consistent: zebrafish embryos and larvae raised in 500 ng L^−1^ to 2 mg L^−1^ PFECHS developed oedema and skeletal malformations at frequencies comparable to those caused by legacy PFOS, although mortality was lower; transcriptomic analysis showed induction of lipid metabolism and detoxification genes, including pparα and cyp1a1 at concentrations near environmental relevance, indicating metabolic imbalance and activation of chemical-stress defences [12]. These invertebrate and vertebrate data together reveal that PFECHS, much like the compounds it was intended to replace, perturbs endocrine function, development, and energy metabolism, even at sub-lethal doses, underscoring the need for extended and multigenerational studies to evaluate population-level impacts.

Our chronic exposure transcriptomic analysis in *D. magna* aligns with and extends this picture. The dominance of metabolic process GO terms among both activated and suppressed BPs indicates broad re-programming of cellular metabolism. Within this context, activation of the “glutathione metabolic process” is notable, as glutathione-dependent antioxidants such as GPX4 protect gametes and pre-implantation embryos from reactive oxygen species; disruption of GPX4 leads to infertility and early embryonic lethality, potentially affecting reproductive functions and early development. Enhanced glutathione metabolism can therefore reflect a compensatory response to oxidative stress that, if overwhelmed, may reduce cell division fidelity and implantation rates at the earliest embryonic stages. Conversely, suppression of the “organonitrogen compound metabolic process” may be linked to gametogenesis because de novo amino-acid synthesis and nitrogen recycling fuel chromatin remodelling and ATP-intensive meiotic stages.

At the CC level, activation of fibre-related terms such as “sarcomere”, “supramolecular fibre”, and “Z disc” suggests that PFECHS affects myofilament organisation. Suppressed CCs, including “protein-containing complex” and “ribonucleoprotein complex”, point to impacts on large macromolecular assemblies. The suppression of cell-structure-associated ontologies such as “membrane-bounded organelle” implies impaired redistribution of the endoplasmic reticulum and mitochondria, which coordinate Ca^2+^ signalling and maternal mRNA storage—processes essential for oocyte maturation and embryonic developmental competence. Conversely, the prevalence of myofilament-related terms among suppressed or perturbed CCs suggests interference with sarcomere assembly; for example, loss of the sarcomere assembly factor Smyd1b disrupts early muscle formation and subsequently lowers developmental and hatching success. These parallels support a scenario in which PFECHS affects muscle development and function alongside reproductive processes.

In both activated and suppressed MFs, enrichment of catalytic activity and ion-binding ontologies indicates broad modification of enzyme-mediated reactions and metal-ion handling. The presence of detoxification-related MFs, such as oxidoreductase and glutathione transferase activities, further points to altered ROS-detoxification pathways that are vital for reproductive health. Suppressed binding-related MFs (e.g., organic cyclic compound binding and nucleic acid binding) may reflect impaired interactions between proteins and hormones, DNA or RNA, potentially altering transcriptional and post-transcriptional regulation of reproduction-related genes. The integrated biological network and orthologous phenotype analysis emphasise that many PFECHS-responsive genes are embedded in reproductive and developmental circuits. The identification of phenotypes such as abnormal developmental rate, abnormal size, altered fertility, and abnormal oxidative stress response in *D. melanogaster* orthologues supports the conclusion that chronic PFECHS exposure can affect both growth and reproductive performance in *D. magna*. The central genes highlighted by network-centrality analysis—UBC9, eIF4A-III, transformer-2 and HDAC1—are all linked to critical reproductive and developmental functions, ranging from specific cellular processes in multicellular organisms to the development of anatomical structures [32,33,34,35]. They are particularly important in gamete generation and development, including oogenesis and the meiotic cycle.

Furthermore, the involvement of the meiotic spindle in chromosome separation and the binding of DEGs to organic cyclic compounds, which may influence hormone function, together with altered regulation of reproduction-related vtg1 and ylk, suggest that interactions with signalling hormones could alter reproduction in *D. magna* because these proteins are essential to the nutrient reserves of developing embryos. Thus, the condensed PFECHS network describes a gene set that is crucial for regulating reproductive health and development and illuminates the genetic interactions that support these biological functions. The prominence of reproductive-related phenotypes such as fertility and of cellular processes such as “female gamete generation” and “female meiotic nuclear division” in the condensed network further suggests that these four central genes may be involved in the reproductive toxicity mechanism of PFECHS in parthenogenetic *D. magna.* The Wnt-based putative AOP for PFECHS offers a mechanistic framework that connects these molecular and phenotypic observations. Because a definitive MIE could not be resolved directly from the transcriptomic evidence, the model assumes that perturbation of CK1ε and CK2 expression acts as a proximal initiating event. Resulting changes in DVL complex formation and TCF/LEF transcriptional activity provide a plausible route to transcriptional dysregulation of genes controlling oocyte maturation and early development, including vtg1, yolkless, and cyclins. At the organismal level, such molecular defects are consistent with the observed suppression of the vitellogenin protein and the lack of overt lethality despite impaired reproductive output. Integrating these findings with oxidative stress signatures—such as up-regulation of glutathione metabolism genes—suggests that PFECHS imposes combined endocrine and redox pressure that may compound over successive generations. Overall, the pathway links CK1ε/CK2-mediated Wnt disruption to transcriptional dysregulation of female reproductive processes and provides a mechanistic basis for evaluating sub-lethal PFAS impacts in freshwater cladocerans.

## 4. Materials and Methods

### 4.1. Transcriptomic Datasets and Preprocessing

Whole microarray data for *D. magna* chronically exposed to TBOEP or PFECHS were retrieved from the NCBI Gene Expression Omnibus (GEO). The TBOEP study corresponds to series GSE55132, profiling animals after a 21-day exposure to 1470 µg L^−1^ on the Agilent 4 × 44 K platform (GPL16592). The PFECHS study corresponds to series GSE75607, generated under identical platform conditions following a 21-day exposure to 6 mg L^−1^.

Raw files were imported into R v4.3.1. Background correction and within array normalisation were performed with the limma package 3.62.2, where multiple probes targeted the same transcript, and signals collapsed to the median intensity. Differential expression was assessed using an empirical Bayes-moderated t statistic; transcripts with |log_2_ fold change| ≥ 1.0 and a Benjamini–Hochberg false discovery rate (FDR) < 0.05 were retained as differentially expressed genes (DEGs).

### 4.2. Functional Enrichment Analysis

Gene ontology (GO) and Kyoto Encyclopaedia of Genes and Genomes (KEGG) term enrichment was performed in clusterProfiler v4.8.1 with the *D. magna* background (taxon 35,525). GO terms (biological process, cellular component, molecular function) with FDR < 0.05 were retained.

### 4.3. Protein Interaction Networks and Hub Gene Mining

Significant DEGs were queried against STRING v12.0 (confidence score > 0.4; “*D. magna*” organism filter). Resulting interaction tables were imported into Cytoscape v3.10.3. Network topologies were analysed by calculating node degree and betweenness centrality scores, and the top 10 ranked genes were defined as hub genes.

### 4.4. Adverse Outcome Pathway (AOP) Inference

Putative molecular key events (MKEs) were anchored to the KEGG pathway ko04310 (Wnt signalling). Candidate stressor/MKE, MKE/MKE and MKE/adverse outcome links were extracted with AOP helpFinder v3.0 (query terms: “TBOEP” or “PFECHS” AND “*Daphnia* OR invertebrate”). Literature co-occurrence scores ≥ 2 were accepted. Final AOP schematics were rendered in Cytoscape v3.10.3. Within this pathway, we prioritised nodes that corresponded to significantly altered transcripts in our DEG lists (e.g., ps-1, CK1ε, CK2), occupied upstream regulatory positions controlling β-catenin stabilisation and TCF/LEF activity, and have published roles in reproduction or development in invertebrates and/or vertebrates. These presenilin- and casein-kinase-associated nodes were therefore treated as putative molecular-initiating events (MIEs) linking the observed transcriptomic changes to downstream reproductive key events, while acknowledging that direct biochemical confirmation in *D. magna* was still lacking.

## 5. Conclusions

EDCs threaten aquatic ecosystems by disturbing hormone-controlled growth and reproduction. Among them, the flame retardants TBOEP and PFECHS now occur in surface waters and persist in wastewater discharge.

Chronic exposure of *D. magna* to high, sub-lethal test concentrations of TBOEP (1470 µg L^−1^) and PFECHS (6 mg L^−1^) in the underlying microarray studies reduced body length and moulting, suppressed vitellogenin, altered cuticle-synthesis genes, and activated detoxification pathways without causing acute lethality. Gene expression profiling (GSE55132, GSE75607) confirmed broad changes in metabolism, cellular processes, and reproduction. Both datasets pointed to Wnt-signal interference and highlighted central hubs UBC9 (LOC116919267) and eIF4A-III (LOC116919853) that bridge oxidative stress and meiotic control. These findings should be interpreted as high-dose mechanistic scenarios that identify susceptible pathways and biomarkers; future research at lower, environmentally realistic concentrations is needed to verify the dose–response relationships and ecological relevance of the proposed AOPs.

Functional enrichment and network analyses allowed the construction of putative adverse outcome pathways (AOPs), linking CK1ε/CK2-mediated Wnt disruption to impaired oogenesis and embryonic development. These molecular events explain how chronic sub-lethal exposure can erode *D. magna* fitness and, by extension, alter plankton dynamics essential for water-column clarity and food-web stability.

Understanding such mechanisms is critical for ecological risk assessment and chemical management. Further studies with time-resolved transcriptomics are needed to gauge mixture effects and to refine water-quality criteria that safeguard freshwater biodiversity and human communities reliant on these ecosystems.

## Figures and Tables

**Table 1:** Top 10 gene ontology terms for up-regulated DEGs and the top 10 GO terms for down-regulated DEGs in *D. magna* subjected to chronic TBOEP exposure.

GO Term Category	Activated GO		Suppressed GO	
	GO Term	FDR	GO Term	FDR
Biological process	Cellular process	0.00018	Biological regulation	2.10 × 10^−3^
	Metabolic process	0.00051	Cellular process	2.10 × 10^−3^
	Organic substance metabolic process	0.00068	Regulation of cellular process	1.13 × 10^−2^
	Primary metabolic process	0.0013	Regulation of biological process	0.0174
	Protein metabolic process	0.0017	Positive regulation of transport	0.0335
	Organonitrogen compound metabolic process	0.0017	Positive regulation of biological process	0.0483
	Macromolecule metabolic process	0.0017	-	-
	Nitrogen compound metabolic process	0.0017		
	Protein localization	0.0043		
	Cellular localization	0.0099		
Cellular component	Cellular anatomical entity	1.8 × 10^−13^	Cellular anatomical entity	6.44 × 10^−8^
	Intracellular anatomical structure	3.16 × 10^−8^	Cytoplasm	3.16 × 10^−6^
	Cytoplasm	1.62 × 10^−7^	Intracellular anatomical structure	1.29 × 10^−5^
	Endomembrane system	0.0016	Intracellular organelle	0.0056
	Membrane-bounded organelle	0.0018	Membrane	0.0072
	Intracellular membrane-bounded organelle	0.0018	-	-
	Intracellular organelle	0.0018		
	Protein-containing complex	0.0053		
	Dendritic shaft	0.0091		
	Cytoplasmic vesicle	0.0096		
Molecular function	Catalytic activity	2.40 × 10^−4^	Binding	0.00033
	Protein binding	5.70 × 10^−3^	-	-
	Catalytic activity, acting on a protein	3.00 × 10^−2^		
	Hydrolase activity	0.03		
	Binding	0.032		

**Table 2:** Endocrine-related gene ontology categories for up-regulated and down-regulated DEGs in *D. magna* following chronic exposure to TBOEP.

Network Type	GO Term Category	GO Term	FDR
Main network	Biological process	Organic substance biosynthetic process	1.56 × 10^−2^
		Organic substance transport	2.21 × 10^−2^
		Anatomical structure development	3.63 × 10^−2^
		Muscle structure development	4.39 × 10^−2^
		Collagen trimer	4.83 × 10^−2^

**Table 3:** Top 10 gene ontology terms for up-regulated DEGs and the top 10 GO terms for down-regulated DEGs in *D. magna* subjected to chronic PFECHS exposure.

GO Term Category	Activated GO		Suppressed GO	
	GO Term	FDR	GO Term	FDR
Biological process	Cellular process	9.71 × 10^−17^	Cellular process	1.98 × 10^−88^
	Metabolic process	4.73 × 10^−11^	Organic substance metabolic process	1.95 × 10^−51^
	Organic substance metabolic process	9.28 × 10^−10^	Metabolic process	2.07 × 10^−51^
	Organonitrogen compound metabolic process	1.26 × 10^−8^	Primary metabolic process	1.23 × 10^−50^
	Response to stimulus	4.84 × 10^−8^	Nitrogen compound metabolic process	5.18 × 10^−50^
	Primary metabolic process	1.23 × 10^−6^	Macromolecule metabolic process	3.06 × 10^−48^
	Nitrogen compound metabolic process	1.40 × 10^−5^	Cellular metabolic process	5.02 × 10^−45^
	Regulation of biological quality	3.83 × 10^−5^	Biological regulation	4.19 × 10^−43^
	Glutathione metabolic process	6.61 × 10^−5^	Cellular component organisation or biogenesis	8.24 × 10^−42^
	Response to ethanol	1.10 × 10^−4^	Regulation of biological process	2.60 × 10^−38^
Cellular component	Cellular anatomical entity	3.54 × 10^−23^	Intracellular anatomical structure	2.37 × 10^−105^
	Cytoplasm	6.45 × 10^−8^	Cellular anatomical entity	1.45 × 10^−96^
	Sarcomere	4.75 × 10^−6^	Intracellular organelle	1.35 × 10^−71^
	Intracellular anatomical structure	5.18 × 10^−6^	Organelle	4.54 × 10^−71^
	Extracellular region	6.61 × 10^−6^	Intracellular membrane-bounded organelle	1.83 × 10^−59^
	Supramolecular fibre	1.10 × 10^−4^	Protein-containing complex	3.80 × 10^−57^
	Membrane	1.10 × 10^−4^	Membrane-bounded organelle	3.80 × 10^−57^
	Z disc	2.30 × 10^−3^	Nucleus	3.64 × 10^−52^
	Organelle	2.90 × 10^−3^	Cytoplasm	6.12 × 10^−50^
	Intracellular organelle	7.60 × 10^−3^	Ribonucleoprotein complex	1.14 × 10^−35^
Molecular function	Catalytic activity	5.48 × 10^−12^	Binding	2.38 × 10^−65^
	Ion binding	1.93 × 10^−7^	Organic cyclic compound binding	6.34 × 10^−49^
	Binding	5.32 × 10^−7^	Heterocyclic compound binding	2.18 × 10^−48^
	Cation binding	5.22 × 10^−5^	Nucleic acid binding	1.03 × 10^−28^
	Oxidoreductase activity	2.10 × 10^−4^	RNA binding	5.49 × 10^−27^
	Glutathione transferase activity	2.50 × 10^−4^	Protein binding	3.23 × 10^−26^
	Metal ion binding	3.00 × 10^−4^	Ion binding	7.42 × 10^−22^
	Transferase activity	6.70 × 10^−4^	Catalytic activity	1.38 × 10^−20^
	Catalytic activity, acting on a protein	2.50 × 10^−3^	Small molecule binding	4.93 × 10^−20^
	Protein binding	2.80 × 10^−3^	Carbohydrate derivative binding	6.75 × 10^−20^

**Table 4:** Endocrine-related gene ontology categories for up-regulated and down-regulated DEGs in *D. magna* following chronic exposure to PFECHS.

Network Type	GO Term Category	GO Term	FDR
Main network	Biological process	Organic substance biosynthetic process	5.44 × 10^−9^
		Developmental process	2.60 × 10^−4^
		Cellular process involved in reproduction in multicellular organism	4.60 × 10^−4^
		Anatomical structure development	5.10 × 10^−4^
		Reproductive process	5.60 × 10^−4^
		Female gamete generation	2.50 × 10^−3^
		Organic substance transport	7.50 × 10^−3^
		Developmental process involved in reproduction	9.40 × 10^−3^
		Multicellular organism development	1.63 × 10^−2^
		Meiotic cell cycle process	1.79 × 10^−2^
		Germ cell development	3.46 × 10^−2^
		Meiotic nuclear division	3.88 × 10^−2^
		Oogenesis	4.01 × 10^−2^
		Female meiotic nuclear division	4.15 × 10^−2^
	Cellular component	Meiotic spindle	1.05 × 10^−2^
	Molecular function	Organic cyclic compound binding	1.41 × 10^−43^

## Data Availability

The original data presented in the study are openly available in NCBI GEO: GSE55132, GSE75607.
